# A Perplexing Case of Pituitary Apoplexy Masquerading as Recurrent Meningitis

**DOI:** 10.1177/2324709618811370

**Published:** 2018-11-15

**Authors:** Madhura Myla, Jeremy Lewis, Alan Beach, Gresa Sylejmani, Mark R. Burge

**Affiliations:** 1 University of New Mexico Health Sciences Center, Albuquerque, NM, USA

**Keywords:** pituitary adenoma, apoplexy, meningitis

## Abstract

In this article, we present an exceptional case of pituitary apoplexy in which a patient presented with meningeal symptoms of headache, stiff neck, and nausea rather than the classical findings of ophthalmoplegia and/or vision loss. The patient has had 2 similar presentations with cerebrospinal fluid showing neutrophilic pleocytosis, as well as a computed tomography scan showing a prominent pituitary gland. On current presentation, the patient’s vital signs were stable and the physical examination was remarkable for nuchal rigidity. Magnetic resonance imaging of the head revealed an expansile pituitary gland lesion measuring 2.0 × 1.7 × 1.5 cm with upward displacement of the overlying optic chiasm. Cerebrospinal fluid showed neutrophilic pleocytosis, low glucose, high protein content, and negative bacterial and fungal cultures. Surgical decompression subsequently revealed findings consistent with pituitary apoplexy. This is the first known case in which a patient had recurrent episodes of meningitis due to pituitary apoplexy in the absence of a clinical deterioration. Early identification of apoplexy masquerading as meningitis will allow early surgical intervention, if necessary, to prevent complications, recurrence, and morbidity. As such, the presence of sterile meningitis in patients with a known pituitary adenoma should be considered for prompt surgical evaluation.

## Introduction

Pituitary apoplexy is a rare clinical syndrome with an incidence ranging from 1% to 26% of pituitary tumors.^1^ There are several published cases of pituitary apoplexy in which the patient presented with sterile meningitis and the diagnosis was made quickly during the first presentation.^2,3^ In this article, we present a case in which the patient presented with recurrent meningitis on multiple occasions without clinical deterioration before the correct diagnosis became apparent.

## Case Presentation

A 59-year-old male with a medical history of hypertension presented to the hospital with headache, stiff neck, and nausea. Past medical history included 2 similar presentations: 21 months prior and 1 month prior. On those occasions, cerebrospinal fluid (CSF) analysis showed neutrophilic pleocytosis, and head computed tomography (CT) scan showed a prominent pituitary gland. The patient was treated empirically for bacterial versus viral meningitis on both occasions. Seventeen months prior, the patient was diagnosed with an apparently nonfunctioning pituitary macroadenoma requiring hormone replacement therapy, but surgical resection of the lesion was not pursued. The relevant laboratory values during that episode include an adrenocorticotropic hormone concentration of 11 pg/mL (reference range = 0-46 pg/mL), a thyroid stimulating hormone concentration of <0.01 mU/L (reference range = 0.5-5.0 mU/L), a growth hormone concentration of 0.16 µg/L (reference range = <5 µg/L), and a prolactin level of 42 ng/mL (reference range = <20 ng/mL). The elevated prolactin level was attributed to pituitary stalk compression. The patient was started on levothyroxine 100 µg by mouth once daily, prednisone 5 mg by mouth once daily, and transdermal testosterone gel 5 g to the skin daily.

During all of these encounters, the review of systems was negative for vision loss, rhinorrhea, rash, penile discharge, or recent travel. The physical examination was significant for nuchal rigidity but negative for Kernig’s, Brudzinski’s, or focal neurological deficits.

On presenting for the third time, the patient was again admitted to the hospital for evaluation and management of presumed acute meningitis. Lumbar puncture with CSF analysis showed neutrophilic pleocytosis (see Table 1) with negative bacterial cultures, and negative viral and fungal studies. Magnetic resonance imaging (MRI) of the brain confirmed the presence of a pituitary macroadenoma, which was unchanged from previous imaging. Given the lack of another explanation for the patient’s recurrent meningitis, a transsphenoidal hypophysectomy was performed, and postoperative histopathological examination confirmed the presence of pituitary apoplexy.

## Laboratory Findings

On initial presentation in the fall of 2015, the CSF was cloudy and colorless with a white blood cell count (WBC) of 4375 cells/mm^3^ (90% neutrophils, 5% lymphocytes), a total protein of 107 mg/dL (reference range = 15-45), a glucose concentration of 43 mg/dL (reference range = 41-84), and a normal plasma glucose level. CSF red blood cells (RBCs) were elevated at 150 cells/mm^3^ (reference range = 0). Bacterial cultures showed no growth. CSF cryptococcal antigen, Venereal Disease Research Laboratory (VDRL) test, cytomegalovirus (CMV) polymerase chain reaction test, QuantiFERON-TB (QN) gold test, West Nile virus antibody, rapid plasma reagin (RPR), Epstein-Barr virus (EBV), enterovirus polymerase chain reaction, and HIV screens were all negative.

In the spring of 2017, CSF was hazy and colorless with a WBC of 1303 cells/mm^3^ (78% neutrophils, 3% lymphocytes), RBC of 33 cells/mm^3^, a glucose concentration of 38 mg/dL, and a total protein of 64 mg/dL. Bacterial cultures were again negative, as were cryptococcal antigen, VDRL, CMV, QN gold, West Nile virus antibody, RPR, EBV, and HIV screens.

On final presentation in the summer of 2017, CSF was hazy and colorless with a WBC of 1469 cells/mm^3^ (78% neutrophils, 7% lymphocytes), a RBC count of 68 cells/mm^3^, a glucose concentration of 39 mg/dL, and a total protein of 112 mg/dL. Bacterial cultures and cryptococcal antigen, VDRL, CMV, QN gold, West Nile virus antibody, RPR, EBV, and HIV screens were again negative. All these laboratory results are summarized in Table 1.

**Table 1.** Laboratory Results From CSF During 3 Successive Admissions for Acute Meningitis.

CSF Analyte	2015 Admission	2016 Admission	2017 Admission	Reference Range
WBC (×10^3^ cells/mm^3^)	4.375	1.303	1.469	0-5
% Neutrophils	90	78	78	0-8
RBC (cells/mm^3^)	150	33	68	0
Glucose (mg/dL)	43	38	39	41-84
Protein (mg/dL)	107	64	112	15-45
Bacterial cultures	No growth	No growth	No growth	No growth
Virus and fungal serologies	Negative	Negative	Negative	Negative

Abbreviations: CSF, cerebrospinal fluid; WBC, while blood cell; RBC, red blood cell.

## Serial Imaging Findings

In 2016, an MRI scan of the brain with and without contrast showed a prominent pituitary gland measuring 1.3 × 1.3 × 1.3 cm with no evidence of acute infarct, hemorrhage, or mass lesion.

In 2017, a CT scan of head showed an expansion of the sella with no acute intracranial hemorrhage. A subsequent MRI showed a sellar and suprasellar lesion with a cystic appearance and marginal contrast enhancement that measured 1.6 × 1.5 × 1.8 cm. There was slight displacement of the optic chiasm and the prechiasmatic optic nerves. A CT cisternogram did not show any CSF leak.

**Figure 1.**
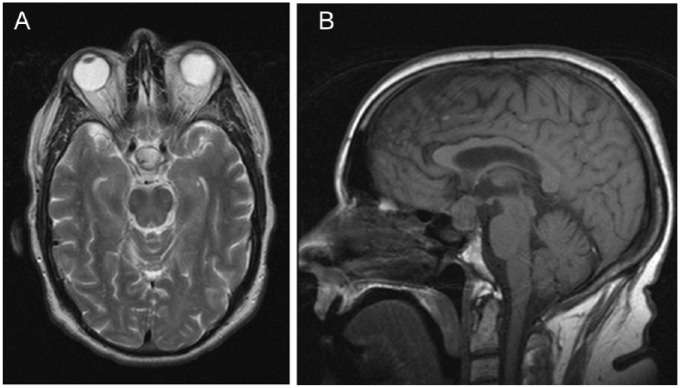
Coronal (A) and sagittal (B) sections of a T2-weighted magnetic resonance image demonstrating a pituitary mass measuring 2.0 × 1.7 × 1.5 cm and extending superiorly with mild displacement of the overlying optic chiasm and optic tracts.

As shown in Figure 1, a preoperative MRI scan demonstrated a 2.0 × 1.7 × 1.5 cm cystic mass in the pituitary gland extending superiorly into the suprasellar cistern with mild displacement of the overlying optic chiasm and optic tracts.

## Treatment

**Figure 2.**
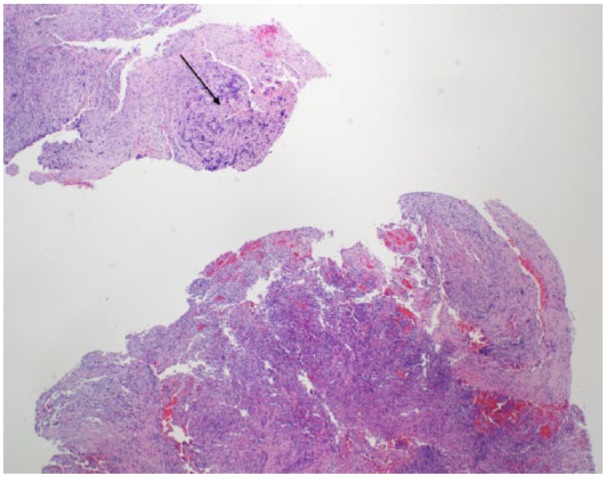
Surgical pathology section of the resected pituitary lesion at 4× magnification, showing necrotic pituitary tissue with hematoxylin-eosin staining. The arrow depicts necrotic pituitary tumor surrounded by acute and chronic inflammatory cells. The presence of blood cells within the pituitary lesion (lower right) confirmed pituitary apoplexy.

Transsphenoidal hypophysectomy was performed and purulent discharge was drained from the sella. As shown in Figure 2, final pathology showed a necrotic pituitary tumor surrounded by acute and chronic inflammatory cells. The presence of blood cells within the pituitary lesion confirmed pituitary apoplexy.

The patient was discharged home in good condition on hydrocortisone (15 mg by mouth every morning and 5 mg by mouth every evening), levothyroxine (100 µg by mouth daily), and transdermal testosterone gel (5 g to skin daily). He was successfully managed on this regimen as an outpatient and did not have any further symptoms of meningitis.

## Discussion

Pituitary apoplexy is a rare clinical syndrome resulting from ischemic or hemorrhagic necrosis of the pituitary gland and with an incidence ranging from 1% to 26% in various studies.^1^ Pituitary apoplexy is characterized by acute headache, eye pain, ophthalmoplegia, vision loss, and pituitary insufficiency. Signs of meningeal irritation, clinically indistinguishable from infectious meningitis, are considered rare.^4,5^ In the setting of pituitary apoplexy, the absence of CSF rhinorrhea does not rule out the possibility of meningitis.

As shown in Table 2, previously published case reports indicate a handful of cases in which pituitary apoplexy presented with sterile meningitis. In those cases, the cause of the sterile meningeal reaction was attributed to the leakage of blood or the expulsion of necrotic tissue into the subarachnoid space. Such leakage induces a cytokine-mediated inflammatory response that results in meningeal irritation and a clinical picture suggestive of acute meningitis.^4,5^

**Table 2.** Previously Reported Cases of Pituitary Apoplexy Mimicking Acute Meningitis.

Report Number	Symptoms	CSF	Pituitary Function	Diagnostic Modality	Intervention	Reference Number
1	Headache, fever, and signs of meningeal irritation	Sterile with 3 lymphocytes/mm and increased protein concentration	Unavailable	MRI	Unavailable	2
2	Nausea, vomiting, fever, headache, and developed left ptosis and CN VI palsy after 3 days	Neutrophilic pleocytosis	Hypopituitarism	MRI	Conservative management	6
3	Headache, vomiting, and diplopia	Sterile lymphocytic pleocytosis	Hypopituitarism	CT scan + clinical signs and symptoms	IV steroids + transsphenoidal resection	7
4	Frontal headache, vomiting, developed bilateral CN VI palsies 1 day later	Mixed pleocytosis with xanthochromia	Hypopituitarism	CT scan + clinical signs and symptoms	IV steroids	7
5	Fever, headache, diplopia, photophobia, and dysarthria	Neutrophilic pleocytosis	Hypopituitarism	CT scan	Data unavailable	8
6	Headache, diplopia, and ophthalmoplegia	Neutrophilic pleocytosis	Hypopituitarism	MRI scan	Transsphenoidal resection	8
7	Headache, nausea, and developed right CN III and VI palsies 2 days later	Neutrophilic pleocytosis	Unavailable	CT scan	Data unavailable	8
8	Fever, headache, neck stiffness, and left CN III and VI palsies	Sterile neutrophilic pleocytosis	Hypopituitarism	MRI scan	Surgical decompression of the sella	9
9	Fever, headache, neck stiffness, and developed right CN III palsy, bitemporal hemianopia 3 days later	Sterile meningitis	Hypopituitarism	MRI scan	Transsphenoidal surgical decompression	10

Abbreviations: CSF, cerebrospinal fluid; MRI, magnetic resonance imaging; CN, cranial nerve; CT, computed tomography; IV, intravenous.

Prior case reports have suggested that conservative medical therapy with glucocorticoid therapy and additional hormone replacement are indicated in mild apoplexy, while surgery involving drainage of the intrasellar hemorrhage and removal of the tumor are indicated in more severe cases, including those that are accompanied by neuro-ophthalmic signs and symptoms, such as altered consciousness, vision loss, and/or ocular palsy.^11-13^ The current case is unique because the patient did not present with the classic findings of apoplexy, but rather with predominant meningeal symptoms, and he had 3 distinct episodes of recurrent meningitis without hemodynamic decompensation.

Table 2 provides a compilation of previously published cases of pituitary apoplexy masquerading as aseptic meningitis. Of the 9 patients shown in Table 2, 8 had ophthalmoplegia, cranial nerve palsy, or diplopia at some point during the hospitalization (ie, cases 2-9). Only one of the patients had a similar presentation to the patient we report here, and none of these cases were characterized by recurrent episodes of meningismus.^2^ Additionally, 7 of the cases from the previously published reports were definitively diagnosed by imaging with either a CT scan or an MRI scan of the brain. Conversely, CT scan and brain MRI were nondiagnostic in the current case, and pituitary apoplexy was not confirmed until postoperative histopathology showed the classical findings of pituitary apoplexy.

Treatment for pituitary apoplexy with progressive neurologic symptoms requires surgical decompression of the sella turcica and resection of the pituitary lesion, and such timely treatment is likely to prevent adverse outcomes.^12^ Accordingly, early neurosurgical evaluation may prove beneficial in patients with a history of pituitary adenoma who present with sterile meningitis. This case highlights the importance of considering pituitary apoplexy in the differential diagnosis of patients presenting with recurrent sterile meningitis. In such cases, early evaluation and treatment may decrease recurrences and reduce or avoid the morbidity and cost burden associated with this condition.
